# Supplementary material for: Store-Operated Ca2+ Entry Is Up-Regulated in Tumour-Infiltrating Lymphocytes from Metastatic Colorectal Cancer Patients
Source: Cancers (Basel). 2022 Jul 7;14(14):3312. doi: 10.3390/cancers14143312 (PMC9315763; doi:10.3390/cancers14143312)
Supplement: Supplementary file 1 [file cancers-14-03312-s001.zip › cancers-1789253-supplementary.pdf]

Supplementary Material

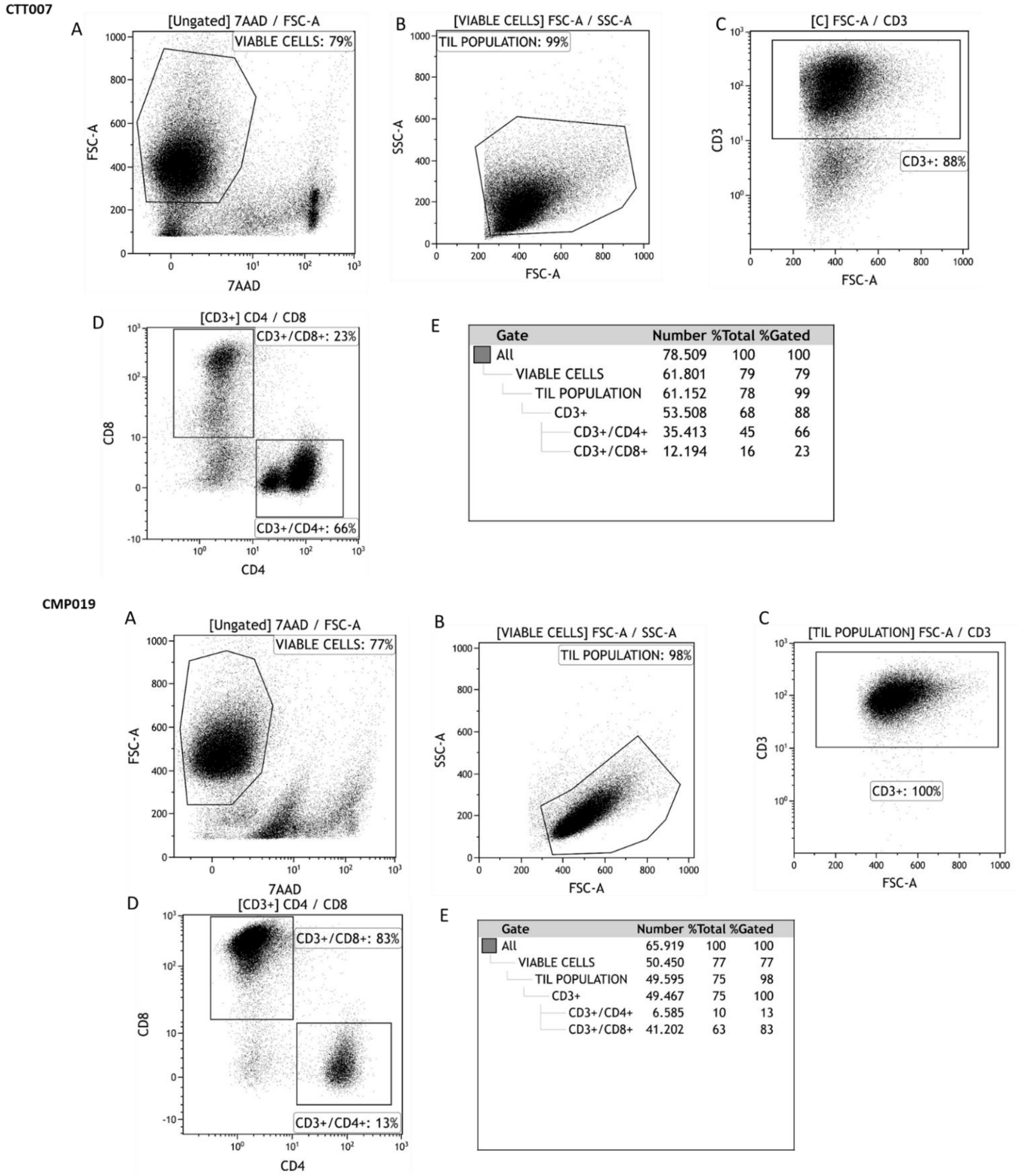

Figure S1. Representative immunophenotype of TILs isolated from mCRC patients. Dot plots of TILs derived from two representative patients (CTT007 and CMP019), in which we documented a preferential expansion of CD3+/CD4+ or CD3+/CD8+ cells, respectively, are shown. For each patient, the panels represent the gating strategy to evaluate TIL cell populations. Panel A shows the percentage of viable cells 7AAD- cells; panel B shows viable TIL cells gated based on physical parameters; panel C shows CD3+ cells present on viable TIL gated cells; panel D shows CD3+/CD4+ and CD3+/CD8+ cells on CD3+ viable TIL gated cells; panel E shows number of cells acquired and gated according to our strategy. .

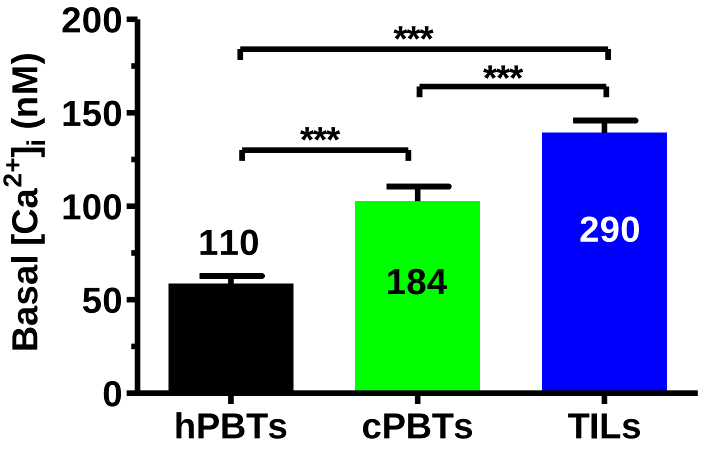

Figure S2. Resting [Ca^{2+}]$_i$ is higher in TILs than in hPBTs and cPBTs. Resting [Ca^{2+}]$_i$ in the three cell types was monitored by using the Grynkiewicz method, as described in [35]. One-way ANOVA followed by the post hoc Bonferroni test: ***$p <$ 0.001. The total number of analysed cells is indicated within the histogram bars.

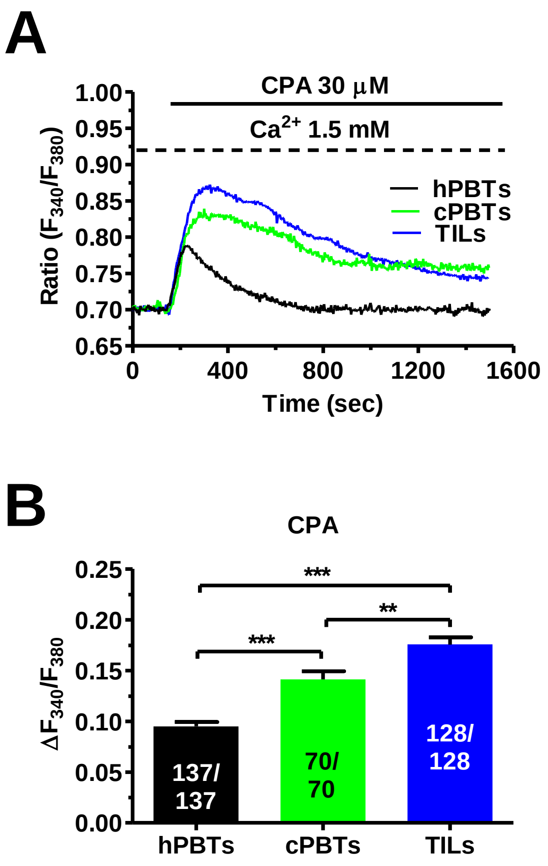

Figure S3. The Ca^{2+} response to CPA is larger in TILs compared to hPBTs and cPBTs. A) Intracellular Ca^{2+} signals evoked by 30 μM CPA in the presence of 1.5 mM Ca^{2+}. Under these conditions, CPA evokes both ER Ca^{2+} release and SOCE activation. B) Mean ± SE of the amplitude CPA-evoked intracellular Ca^{2+} mobilisation. One-way ANOVA followed by the post hoc Bonferroni test: $**p < 0.01$ and $***p < 0.001$. The numbers within or above the histogram bars indicate the number of responding cells over the total number of analysed cells.

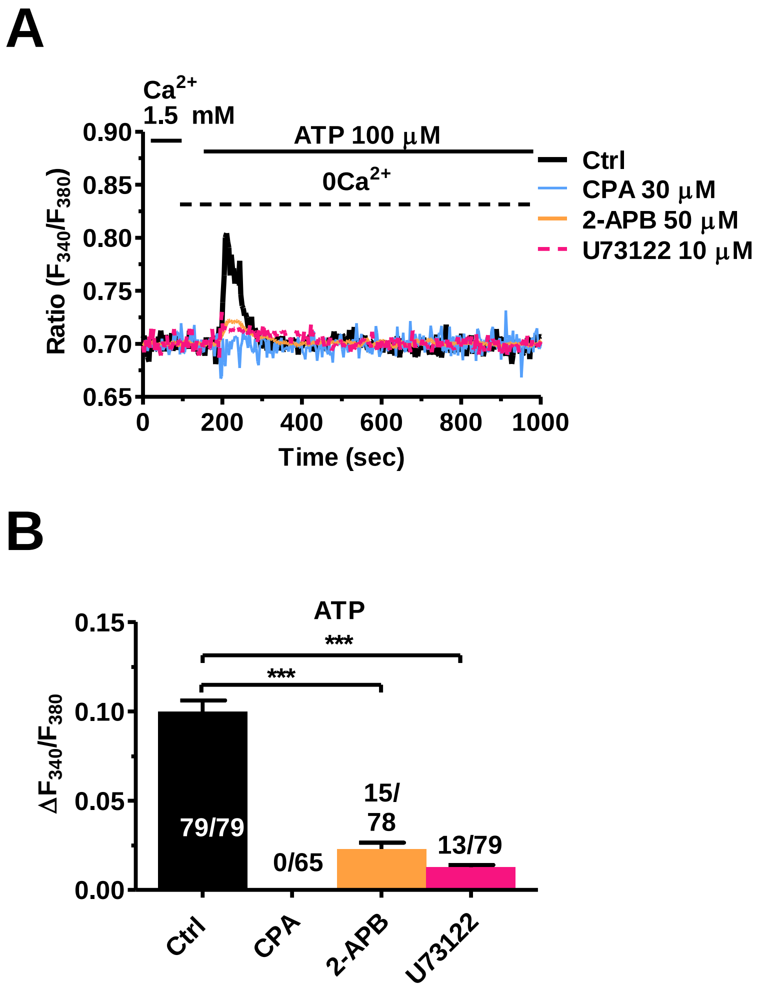

Figure S4. Evidence that InsP$_3$Rs mediate ATP-evoked intracellular Ca^{2+} release in hPBTs. A) Intracellular Ca^{2+} release was evoked by the InsP$_3$-producing autacoid, ATP (100 μM), in the absence (Ctrl) and presence of the following drugs: U73122 (10 μM, 20 min), which inhibits basal InsP$_3$ production by interfering with PLC activity; 2-APB (50 μM 30 min), which blocks InsP$_3$Rs; and CPA (30 μM, 20 min), which specifically depletes the ER Ca^{2+} content. B) Mean ± SE of the amplitude ATP-evoked intracellular Ca^{2+} mobilisation under the designated treatments. One-way ANOVA followed by the post hoc Bonferroni test: ***$p < 0.001$. The numbers within or above the histogram bars indicate the number of responding cells over the total number of analysed cells.

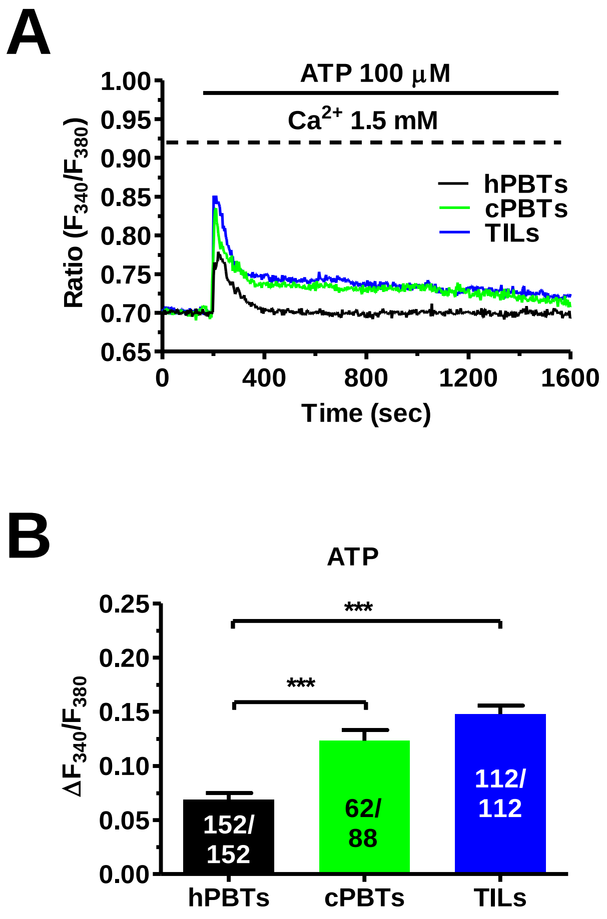

Figure S5. The Ca^{2+} response to ATP is larger in TILs than in hPBTs and cPBTs. A) Intracellular Ca^{2+} signals evoked by 100 µM ATP in the presence of 1.5 mM Ca^{2+}. Under these conditions, ATP evokes both ER Ca^{2+} release and SOCE activation. B) Mean ± SE of the amplitude ATP-evoked intracellular Ca^{2+} mobilisation. One-way ANOVA followed by the post hoc Bonferroni test: ***p* < 0.01 and ****p* < 0.001. The numbers within or above the histogram bars indicate the number of responding cells over the total number of analysed cells.

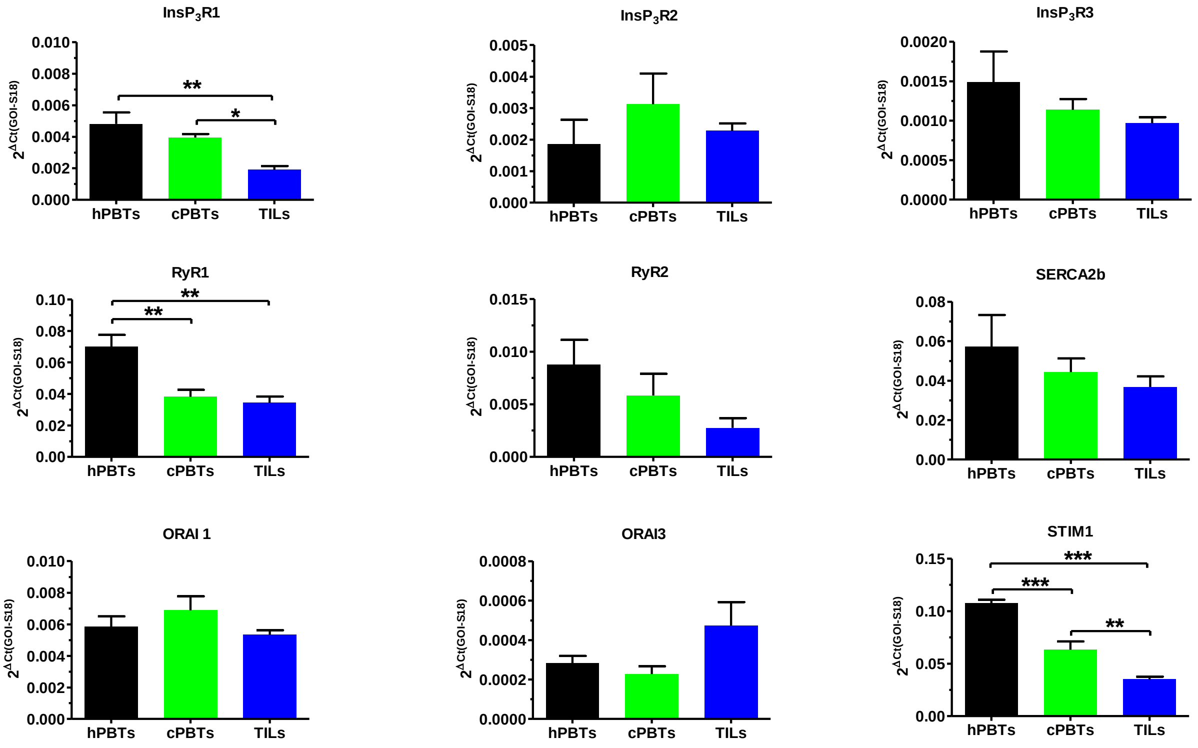

Figure S6. Differential expression of the main component of the Ca^{2+}-handling machinery in hPBTs, cPBTs and TILs. Comparison of the mRNA levels of InsP3R1, InsP3R2, InsP3R3, RyR1, RyR2, SERCA2B, ORAI1, ORAI3 and STIM1 in hPBTs, cPBTs and TILs. Data are expressed as mean ± SE of three qPCR runs performed in triplicate using samples prepared from four healthy subjects (hPBTs) and four patients with mCRC (cPBTs and TILs). One-way ANOVA followed by the post hoc Bonferroni test: *$p < 0.05$, **$p < 0.01$, and ***$p < 0.001$.

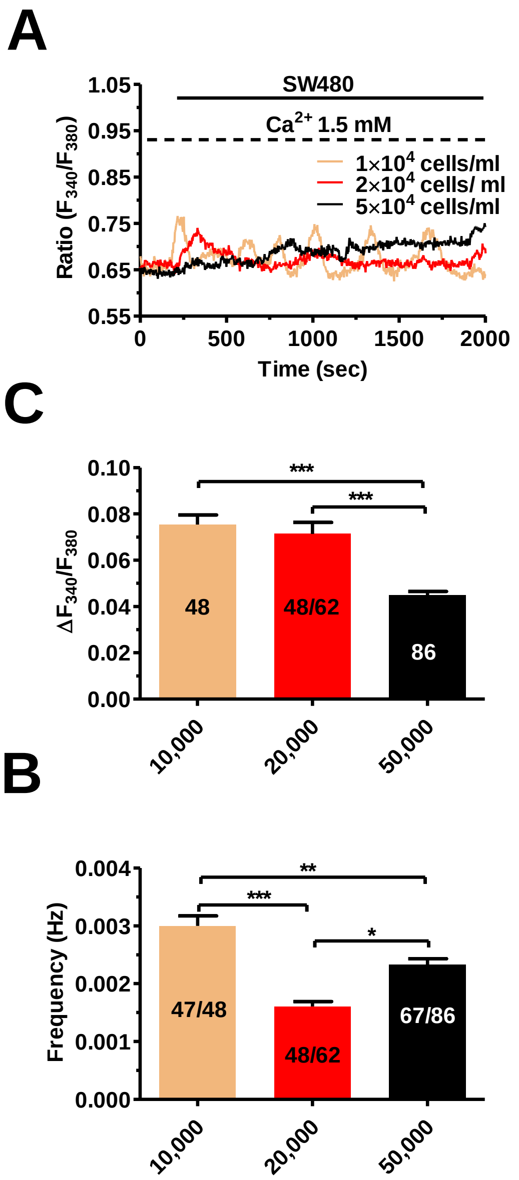

Figure S7. Intracellular Ca^{2+} oscillations elicited by SW480 cells in TILs. A) Intracellular Ca^{2+} oscillations evoked by TIL exposure to different doses of SW480 cells, which represent a widely employed immortalised mCRC cell line. B) Mean ± SD of oscillation frequency in TILs challenged with different doses of SW480 cells. One-way ANOVA followed by the post hoc Bonferroni test: ***$p < 0.001$. C) Mean ± SD of the amplitude of the 1st Ca^{2+} spike in TILs challenged to different doses of SW480 cells. One-way ANOVA followed by the post hoc Bonferroni test: *$p < 0.05$, **$p < 0.01$ and ***$p < 0.001$. In panels B and C, the numbers within or above the histogram bars indicate the number of responding cells over the total number of analysed cells.

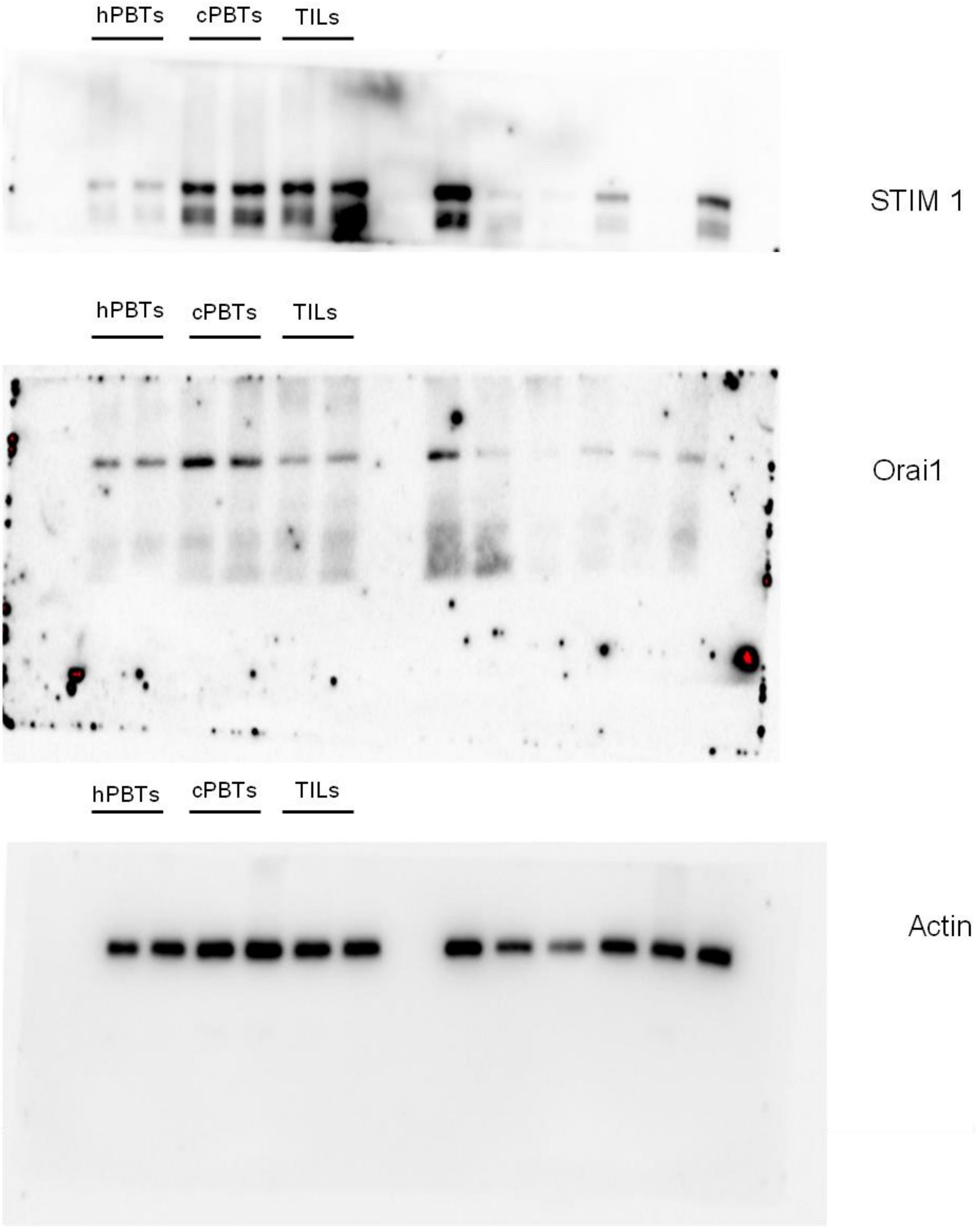

Figure S8. Whole Western blot showing all bands depicted in Figure 4B.

Table S1. Clinical characteristics of patients.

UPN	Age/sex	Disease	Source of tumour cells	Pathology
CTT007	78/F	mCRC	Liver metastasis*	Adenocarcinoma (Cytokeratin 20+; CDX2+)
BNZ008	67/M	mCRC	Liver metastasis*	Adenocarcinoma (Cytokeratin 20+; CDX2+)
NSL009	67/M	mCRC	Liver metastasis*	Adenocarcinoma
FRV010	79/F	mCRC	Liver metastasis*	Adenocarcinoma (Cytokeratin 20+; CDX2+)
MRN014	54/F	mCRC	Liver metastasis*	Adenocarcinoma (Cytokeratin 20+; CDX2+)
PLT016	59/M	mCRC	Liver metastasis*	Adenocarcinoma
GRN017	55/F	mCRC	Liver metastasis*	Adenocarcinoma KRAS+
CMP019	75/M	mCRC	Liver metastasis*	Adenocarcinoma (Cytokeratin 20+; CDX2+)
MRT020	49/M	mCRC	Liver metastasis*	Adenocarcinoma (CDX2+)
DMR021	77/F	mCRC	Liver metastasis*	Adenocarcinoma (CDX2+)

Five males and five females affected by mCRC who underwent surgery for liver metastasis were included in the study. Among the patients initially enrolled, we included study patients from which we were able to obtain enough TILs and cPBTs to accomplish the aims of the study, after having set up the best methodological approach for the ex vivo expansion of the TILs. * 80% tumour viability.

Table S2. List of oligonucleotide primers used for real-time PCR.

**Protein Name**	**Gene Name**	**Forward/reverse (5'→3')**	**Accession No.**
S18	RPS18	TGCGAGTACTCAACACCAACA<br>CTGCTTTCCTCAACACCACA	NM_022551
ORAI1	ORAI1	GACCTCGGCTCTGCTCTC<br>TGATCATGAGCGCAAACAGG	NM_032790
ORAI2	ORAI2	CCCTCCTCTCCGGCTTTG<br>TGATGAGGAGGGCGAACAG	NM_001126340
ORAI3	ORAI3	ACGTCTGCCTTGCTCTCG<br>ACCATGAGTGCAAAGAGGTG	NM_152288
STIM1	STIM1	GCCTCAGCCATAGTCACAGT<br>ATGTTACGGACTGCCTCGAA	NM_001277961
STIM2	STIM2	GACGGATGCGAGCTTGTG<br>AAGCATGGTGGACTCAGTGA	NM_001169117
$InsP_3R1$	ITPR1	AAGAAGGGCAGAAGGAGGAC<br>TTCGTTGATGGCCAGGTATT	NM_002222
$InsP_3R2$	ITPR2	AATGTGACCATGGGGATGAT<br>CGATCCTGGTTTGAGCATCT	NM_002223
$InsP_3R3$	ITPR3	TTATGCAGTTTCGGGACCAC<br>TTGCCCTTGTACTCGTCACA	NM_002224
RyR1	RYR1	CTTCCTGGAGCCCACTAGC<br>CGTGTTAGCCAGCATCTCCT	NM_000540
RyR2	RYR2	CCGCAACCATCCACAAAGAA<br>AAGGTGCAGATGGAGAGGTC	NM_001035
RyR3	RYR3	CGCCACCATTCATAAGGAGC<br>GGGCTCTGACAGATAGGGAC	NM_001036
SERCA2b	ATP2A2,<br>variant b	AATGTGTAACGCCCTCAACA<br>GCAGGCTGCACACACTCTT	NM_170665

Table S3. List of primary antibodies used to characterise the SOCE machinery.

Primary antibody	Dilution	Cat. N.	Source
Anti-STIM 1	1:500	4916	Cell signalling
Anti-Orai1	1:500	PA-74181	Invitrogen
Anti-β-actin	1:2000	A1978	Sigma-Aldrich
